# Supplementary material for: HIVprotI: an integrated web based platform for prediction and design of HIV proteins inhibitors
Source: J Cheminform. 2018 Mar 9;10:12. doi: 10.1186/s13321-018-0266-y (PMC5845081; doi:10.1186/s13321-018-0266-y)
Supplement: Supplementary file 2 — Additional file 2. Source code of HIVProtI web server. [file 13321_2018_266_MOESM2_ESM.zip › HIVprotI_Source-code/hivprotI/weka-3-6-6/doc/weka/classifiers/meta/ThresholdSelector.html]

ThresholdSelector


---


|  |  |  |
| --- | --- | --- |
| **Overview** \| **Package** \| **Class** \| **Tree** \| **Deprecated** \| **Index** \| **Help** \| **Weka's home** | |  |
| **PREV CLASS**   **NEXT CLASS** | **FRAMES**    **NO FRAMES**     **All Classes** |  |
| SUMMARY: NESTED \| FIELD \| CONSTR \| METHOD | DETAIL: FIELD \| CONSTR \| METHOD |  |


---


## weka.classifiers.meta Class ThresholdSelector

```
java.lang.Object
  weka.classifiers.Classifier
      weka.classifiers.SingleClassifierEnhancer
          weka.classifiers.RandomizableSingleClassifierEnhancer
              weka.classifiers.meta.ThresholdSelector
```

**All Implemented Interfaces:**: java.io.Serializable, java.lang.Cloneable, CapabilitiesHandler, Drawable, OptionHandler, Randomizable, RevisionHandler

---

``` public class ThresholdSelector extends RandomizableSingleClassifierEnhancer implements OptionHandler, Drawable ```

A metaclassifier that selecting a mid-point threshold on the probability output by a Classifier. The midpoint threshold is set so that a given performance measure is optimized. Currently this is the F-measure. Performance is measured either on the training data, a hold-out set or using cross-validation. In addition, the probabilities returned by the base learner can have their range expanded so that the output probabilities will reside between 0 and 1 (this is useful if the scheme normally produces probabilities in a very narrow range).


Valid options are:

```
 -C <integer>
  The class for which threshold is determined. Valid values are:
  1, 2 (for first and second classes, respectively), 3 (for whichever
  class is least frequent), and 4 (for whichever class value is most
  frequent), and 5 (for the first class named any of "yes","pos(itive)"
  "1", or method 3 if no matches). (default 5).
```

```
 -X <number of folds>
  Number of folds used for cross validation. If just a
  hold-out set is used, this determines the size of the hold-out set
  (default 3).
```

```
 -R <integer>
  Sets whether confidence range correction is applied. This
  can be used to ensure the confidences range from 0 to 1.
  Use 0 for no range correction, 1 for correction based on
  the min/max values seen during threshold selection
  (default 0).
```

```
 -E <integer>
  Sets the evaluation mode. Use 0 for
  evaluation using cross-validation,
  1 for evaluation using hold-out set,
  and 2 for evaluation on the
  training data (default 1).
```

```
 -M [FMEASURE|ACCURACY|TRUE_POS|TRUE_NEG|TP_RATE|PRECISION|RECALL]
  Measure used for evaluation (default is FMEASURE).
```

```
 -manual <real>
  Set a manual threshold to use. This option overrides
  automatic selection and options pertaining to
  automatic selection will be ignored.
  (default -1, i.e. do not use a manual threshold).
```

```
 -S <num>
  Random number seed.
  (default 1)
```

```
 -D
  If set, classifier is run in debug mode and
  may output additional info to the console
```

```
 -W
  Full name of base classifier.
  (default: weka.classifiers.functions.Logistic)
```

```
 Options specific to classifier weka.classifiers.functions.Logistic:
```

```
 -D
  Turn on debugging output.
```

```
 -R <ridge>
  Set the ridge in the log-likelihood.
```

```
 -M <number>
  Set the maximum number of iterations (default -1, until convergence).
```

Options after -- are passed to the designated sub-classifier.

**Version:**
:   $Revision: 1.43 $

**Author:**
:   Eibe Frank (eibe@cs.waikato.ac.nz)

**See Also:**: Serialized Form

---

| **Field Summary** | |
| --- | --- |
| `static int` | `ACCURACY`             accuracy |
| `static int` | `EVAL_CROSS_VALIDATION`             n-fold cross-validation |
| `static int` | `EVAL_TRAINING_SET`             entire training set |
| `static int` | `EVAL_TUNED_SPLIT`             single tuned fold |
| `static int` | `FMEASURE`             F-measure |
| `static int` | `OPTIMIZE_0`             first class value |
| `static int` | `OPTIMIZE_1`             second class value |
| `static int` | `OPTIMIZE_LFREQ`             least frequent class value |
| `static int` | `OPTIMIZE_MFREQ`             most frequent class value |
| `static int` | `OPTIMIZE_POS_NAME`             class value name, either 'yes' or 'pos(itive)' |
| `static int` | `PRECISION`             precision |
| `static int` | `RANGE_BOUNDS`             Correct based on min/max observed |
| `static int` | `RANGE_NONE`             no range correction |
| `static int` | `RECALL`             recall |
| `static Tag[]` | `TAGS_EVAL`             The evaluation modes |
| `static Tag[]` | `TAGS_MEASURE`             the measure to use |
| `static Tag[]` | `TAGS_OPTIMIZE`             How to determine which class value to optimize for |
| `static Tag[]` | `TAGS_RANGE`             Type of correction applied to threshold range |
| `static int` | `TP_RATE`             true-positive rate |
| `static int` | `TRUE_NEG`             true-negative |
| `static int` | `TRUE_POS`             true-positive |

**Fields inherited from interface weka.core.Drawable**

`BayesNet, Newick, NOT_DRAWABLE, TREE`


**Constructor Summary**

`ThresholdSelector()`
  
          Constructor.


| **Method Summary** | |
| --- | --- |
| `void` | `buildClassifier(Instances instances)`             Generates the classifier. |
| `java.lang.String` | `designatedClassTipText()` |
| `double[]` | `distributionForInstance(Instance instance)`             Calculates the class membership probabilities for the given test instance. |
| `java.lang.String` | `evaluationModeTipText()` |
| `Capabilities` | `getCapabilities()`             Returns default capabilities of the classifier. |
| `SelectedTag` | `getDesignatedClass()`             Gets the method to determine which class value to optimize. |
| `SelectedTag` | `getEvaluationMode()`             Gets the evaluation mode used. |
| `double` | `getManualThresholdValue()`             Returns the value of the manual threshold. |
| `SelectedTag` | `getMeasure()`             get measure used for determining threshold |
| `int` | `getNumXValFolds()`             Get the number of folds used for cross-validation. |
| `java.lang.String[]` | `getOptions()`             Gets the current settings of the Classifier. |
| `SelectedTag` | `getRangeCorrection()`             Gets the confidence range correction mode used. |
| `java.lang.String` | `getRevision()`             Returns the revision string. |
| `java.lang.String` | `globalInfo()` |
| `java.lang.String` | `graph()`             Returns graph describing the classifier (if possible). |
| `int` | `graphType()`             Returns the type of graph this classifier represents. |
| `java.util.Enumeration` | `listOptions()`             Returns an enumeration describing the available options. |
| `static void` | `main(java.lang.String[] argv)`             Main method for testing this class. |
| `java.lang.String` | `manualThresholdValueTipText()` |
| `java.lang.String` | `measureTipText()`             Tooltip for this property. |
| `java.lang.String` | `numXValFoldsTipText()` |
| `java.lang.String` | `rangeCorrectionTipText()` |
| `void` | `setDesignatedClass(SelectedTag newMethod)`             Sets the method to determine which class value to optimize. |
| `void` | `setEvaluationMode(SelectedTag newMethod)`             Sets the evaluation mode used. |
| `void` | `setManualThresholdValue(double threshold)`             Sets the value for a manual threshold. |
| `void` | `setMeasure(SelectedTag newMeasure)`             set measure used for determining threshold |
| `void` | `setNumXValFolds(int newNumFolds)`             Set the number of folds used for cross-validation. |
| `void` | `setOptions(java.lang.String[] options)`             Parses a given list of options. |
| `void` | `setRangeCorrection(SelectedTag newMethod)`             Sets the confidence range correction mode used. |
| `java.lang.String` | `toString()`             Returns description of the cross-validated classifier. |

**Methods inherited from class weka.classifiers.RandomizableSingleClassifierEnhancer**

`getSeed, seedTipText, setSeed`

**Methods inherited from class weka.classifiers.SingleClassifierEnhancer**

`classifierTipText, getClassifier, setClassifier`

**Methods inherited from class weka.classifiers.Classifier**

`classifyInstance, debugTipText, forName, getDebug, makeCopies, makeCopy, setDebug`

**Methods inherited from class java.lang.Object**

`equals, getClass, hashCode, notify, notifyAll, wait, wait, wait`

**Field Detail**

### RANGE\_NONE

```
public static final int RANGE_NONE
```

:   no range correction

    **See Also:**: Constant Field Values

---


### RANGE\_BOUNDS

```
public static final int RANGE_BOUNDS
```

:   Correct based on min/max observed

    **See Also:**: Constant Field Values

---


### TAGS\_RANGE

```
public static final Tag[] TAGS_RANGE
```

:   Type of correction applied to threshold range

---


### EVAL\_TRAINING\_SET

```
public static final int EVAL_TRAINING_SET
```

:   entire training set

    **See Also:**: Constant Field Values

---


### EVAL\_TUNED\_SPLIT

```
public static final int EVAL_TUNED_SPLIT
```

:   single tuned fold

    **See Also:**: Constant Field Values

---


### EVAL\_CROSS\_VALIDATION

```
public static final int EVAL_CROSS_VALIDATION
```

:   n-fold cross-validation

    **See Also:**: Constant Field Values

---


### TAGS\_EVAL

```
public static final Tag[] TAGS_EVAL
```

:   The evaluation modes

---


### OPTIMIZE\_0

```
public static final int OPTIMIZE_0
```

:   first class value

    **See Also:**: Constant Field Values

---


### OPTIMIZE\_1

```
public static final int OPTIMIZE_1
```

:   second class value

    **See Also:**: Constant Field Values

---


### OPTIMIZE\_LFREQ

```
public static final int OPTIMIZE_LFREQ
```

:   least frequent class value

    **See Also:**: Constant Field Values

---


### OPTIMIZE\_MFREQ

```
public static final int OPTIMIZE_MFREQ
```

:   most frequent class value

    **See Also:**: Constant Field Values

---


### OPTIMIZE\_POS\_NAME

```
public static final int OPTIMIZE_POS_NAME
```

:   class value name, either 'yes' or 'pos(itive)'

    **See Also:**: Constant Field Values

---


### TAGS\_OPTIMIZE

```
public static final Tag[] TAGS_OPTIMIZE
```

:   How to determine which class value to optimize for

---


### FMEASURE

```
public static final int FMEASURE
```

:   F-measure

    **See Also:**: Constant Field Values

---


### ACCURACY

```
public static final int ACCURACY
```

:   accuracy

    **See Also:**: Constant Field Values

---


### TRUE\_POS

```
public static final int TRUE_POS
```

:   true-positive

    **See Also:**: Constant Field Values

---


### TRUE\_NEG

```
public static final int TRUE_NEG
```

:   true-negative

    **See Also:**: Constant Field Values

---


### TP\_RATE

```
public static final int TP_RATE
```

:   true-positive rate

    **See Also:**: Constant Field Values

---


### PRECISION

```
public static final int PRECISION
```

:   precision

    **See Also:**: Constant Field Values

---


### RECALL

```
public static final int RECALL
```

:   recall

    **See Also:**: Constant Field Values

---


### TAGS\_MEASURE

```
public static final Tag[] TAGS_MEASURE
```

:   the measure to use


**Constructor Detail**

### ThresholdSelector

```
public ThresholdSelector()
```

:   Constructor.


**Method Detail**

### measureTipText

```
public java.lang.String measureTipText()
```

:   Tooltip for this property.

    :   **Returns:**: tip text for this property suitable for displaying in the explorer/experimenter gui

---


### setMeasure

```
public void setMeasure(SelectedTag newMeasure)
```

:   set measure used for determining threshold

    :   **Parameters:**: `newMeasure` - Tag representing measure to be used

---


### getMeasure

```
public SelectedTag getMeasure()
```

:   get measure used for determining threshold

    :   **Returns:**: Tag representing measure used

---


### listOptions

```
public java.util.Enumeration listOptions()
```

:   Returns an enumeration describing the available options.

    :   **Specified by:**: `listOptions` in interface `OptionHandler` **Overrides:**: `listOptions` in class `RandomizableSingleClassifierEnhancer`
    :   **Returns:**: an enumeration of all the available options.

---


### setOptions

```
public void setOptions(java.lang.String[] options)
                throws java.lang.Exception
```

:   Parses a given list of options.


    Valid options are:

    ```
     -C <integer>
      The class for which threshold is determined. Valid values are:
      1, 2 (for first and second classes, respectively), 3 (for whichever
      class is least frequent), and 4 (for whichever class value is most
      frequent), and 5 (for the first class named any of "yes","pos(itive)"
      "1", or method 3 if no matches). (default 5).
    ```

    ```
     -X <number of folds>
      Number of folds used for cross validation. If just a
      hold-out set is used, this determines the size of the hold-out set
      (default 3).
    ```

    ```
     -R <integer>
      Sets whether confidence range correction is applied. This
      can be used to ensure the confidences range from 0 to 1.
      Use 0 for no range correction, 1 for correction based on
      the min/max values seen during threshold selection
      (default 0).
    ```

    ```
     -E <integer>
      Sets the evaluation mode. Use 0 for
      evaluation using cross-validation,
      1 for evaluation using hold-out set,
      and 2 for evaluation on the
      training data (default 1).
    ```

    ```
     -M [FMEASURE|ACCURACY|TRUE_POS|TRUE_NEG|TP_RATE|PRECISION|RECALL]
      Measure used for evaluation (default is FMEASURE).
    ```

    ```
     -manual <real>
      Set a manual threshold to use. This option overrides
      automatic selection and options pertaining to
      automatic selection will be ignored.
      (default -1, i.e. do not use a manual threshold).
    ```

    ```
     -S <num>
      Random number seed.
      (default 1)
    ```

    ```
     -D
      If set, classifier is run in debug mode and
      may output additional info to the console
    ```

    ```
     -W
      Full name of base classifier.
      (default: weka.classifiers.functions.Logistic)
    ```

    ```
     Options specific to classifier weka.classifiers.functions.Logistic:
    ```

    ```
     -D
      Turn on debugging output.
    ```

    ```
     -R <ridge>
      Set the ridge in the log-likelihood.
    ```

    ```
     -M <number>
      Set the maximum number of iterations (default -1, until convergence).
    ```

    Options after -- are passed to the designated sub-classifier.

    :   **Specified by:**: `setOptions` in interface `OptionHandler` **Overrides:**: `setOptions` in class `RandomizableSingleClassifierEnhancer`
    :   **Parameters:**: `options` - the list of options as an array of strings **Throws:**: `java.lang.Exception` - if an option is not supported

---


### getOptions

```
public java.lang.String[] getOptions()
```

:   Gets the current settings of the Classifier.

    :   **Specified by:**: `getOptions` in interface `OptionHandler` **Overrides:**: `getOptions` in class `RandomizableSingleClassifierEnhancer`
    :   **Returns:**: an array of strings suitable for passing to setOptions

---


### getCapabilities

```
public Capabilities getCapabilities()
```

:   Returns default capabilities of the classifier.

    :   **Specified by:**: `getCapabilities` in interface `CapabilitiesHandler` **Overrides:**: `getCapabilities` in class `SingleClassifierEnhancer`
    :   **Returns:**: the capabilities of this classifier **See Also:**: `Capabilities`

---


### buildClassifier

```
public void buildClassifier(Instances instances)
                     throws java.lang.Exception
```

:   Generates the classifier.

    :   **Specified by:**: `buildClassifier` in class `Classifier`
    :   **Parameters:**: `instances` - set of instances serving as training data **Throws:**: `java.lang.Exception` - if the classifier has not been generated successfully

---


### distributionForInstance

```
public double[] distributionForInstance(Instance instance)
                                 throws java.lang.Exception
```

:   Calculates the class membership probabilities for the given test instance.

    :   **Overrides:**: `distributionForInstance` in class `Classifier`
    :   **Parameters:**: `instance` - the instance to be classified **Returns:**: predicted class probability distribution **Throws:**: `java.lang.Exception` - if instance could not be classified successfully

---


### globalInfo

```
public java.lang.String globalInfo()
```

:   **Returns:**: a description of the classifier suitable for displaying in the explorer/experimenter gui

---


### designatedClassTipText

```
public java.lang.String designatedClassTipText()
```

:   **Returns:**: tip text for this property suitable for displaying in the explorer/experimenter gui

---


### getDesignatedClass

```
public SelectedTag getDesignatedClass()
```

:   Gets the method to determine which class value to optimize. Will
    be one of OPTIMIZE\_0, OPTIMIZE\_1, OPTIMIZE\_LFREQ, OPTIMIZE\_MFREQ,
    OPTIMIZE\_POS\_NAME.

    :   **Returns:**: the class selection mode.

---


### setDesignatedClass

```
public void setDesignatedClass(SelectedTag newMethod)
```

:   Sets the method to determine which class value to optimize. Will
    be one of OPTIMIZE\_0, OPTIMIZE\_1, OPTIMIZE\_LFREQ, OPTIMIZE\_MFREQ,
    OPTIMIZE\_POS\_NAME.

    :   **Parameters:**: `newMethod` - the new class selection mode.

---


### evaluationModeTipText

```
public java.lang.String evaluationModeTipText()
```

:   **Returns:**: tip text for this property suitable for displaying in the explorer/experimenter gui

---


### setEvaluationMode

```
public void setEvaluationMode(SelectedTag newMethod)
```

:   Sets the evaluation mode used. Will be one of
    EVAL\_TRAINING, EVAL\_TUNED\_SPLIT, or EVAL\_CROSS\_VALIDATION

    :   **Parameters:**: `newMethod` - the new evaluation mode.

---


### getEvaluationMode

```
public SelectedTag getEvaluationMode()
```

:   Gets the evaluation mode used. Will be one of
    EVAL\_TRAINING, EVAL\_TUNED\_SPLIT, or EVAL\_CROSS\_VALIDATION

    :   **Returns:**: the evaluation mode.

---


### rangeCorrectionTipText

```
public java.lang.String rangeCorrectionTipText()
```

:   **Returns:**: tip text for this property suitable for displaying in the explorer/experimenter gui

---


### setRangeCorrection

```
public void setRangeCorrection(SelectedTag newMethod)
```

:   Sets the confidence range correction mode used. Will be one of
    RANGE\_NONE, or RANGE\_BOUNDS

    :   **Parameters:**: `newMethod` - the new correciton mode.

---


### getRangeCorrection

```
public SelectedTag getRangeCorrection()
```

:   Gets the confidence range correction mode used. Will be one of
    RANGE\_NONE, or RANGE\_BOUNDS

    :   **Returns:**: the confidence correction mode.

---


### numXValFoldsTipText

```
public java.lang.String numXValFoldsTipText()
```

:   **Returns:**: tip text for this property suitable for displaying in the explorer/experimenter gui

---


### getNumXValFolds

```
public int getNumXValFolds()
```

:   Get the number of folds used for cross-validation.

    :   **Returns:**: the number of folds used for cross-validation.

---


### setNumXValFolds

```
public void setNumXValFolds(int newNumFolds)
```

:   Set the number of folds used for cross-validation.

    :   **Parameters:**: `newNumFolds` - the number of folds used for cross-validation.

---


### graphType

```
public int graphType()
```

:   Returns the type of graph this classifier
    represents.

    :   **Specified by:**: `graphType` in interface `Drawable`
    :   **Returns:**: the type of graph this classifier represents

---


### graph

```
public java.lang.String graph()
                       throws java.lang.Exception
```

:   Returns graph describing the classifier (if possible).

    :   **Specified by:**: `graph` in interface `Drawable`
    :   **Returns:**: the graph of the classifier in dotty format **Throws:**: `java.lang.Exception` - if the classifier cannot be graphed

---


### manualThresholdValueTipText

```
public java.lang.String manualThresholdValueTipText()
```

:   **Returns:**: tip text for this property suitable for displaying in the explorer/experimenter gui

---


### setManualThresholdValue

```
public void setManualThresholdValue(double threshold)
                             throws java.lang.Exception
```

:   Sets the value for a manual threshold. If this option
    is set (non-negative value between 0 and 1), then options
    pertaining to automatic threshold selection are ignored.

    :   **Parameters:**: `threshold` - the manual threshold to use **Throws:**: `java.lang.Exception`

---


### getManualThresholdValue

```
public double getManualThresholdValue()
```

:   Returns the value of the manual threshold. (a negative
    value indicates that no manual threshold is being used.

    :   **Returns:**: the value of the manual threshold.

---


### toString

```
public java.lang.String toString()
```

:   Returns description of the cross-validated classifier.

    :   **Overrides:**: `toString` in class `java.lang.Object`
    :   **Returns:**: description of the cross-validated classifier as a string

---


### getRevision

```
public java.lang.String getRevision()
```

:   Returns the revision string.

    :   **Specified by:**: `getRevision` in interface `RevisionHandler` **Overrides:**: `getRevision` in class `Classifier`
    :   **Returns:**: the revision

---


### main

```
public static void main(java.lang.String[] argv)
```

:   Main method for testing this class.

    :   **Parameters:**: `argv` - the options


---


|  |  |  |
| --- | --- | --- |
| **Overview** \| **Package** \| **Class** \| **Tree** \| **Deprecated** \| **Index** \| **Help** \| **Weka's home** | |  |
| **PREV CLASS**   **NEXT CLASS** | **FRAMES**    **NO FRAMES**     **All Classes** |  |
| SUMMARY: NESTED \| FIELD \| CONSTR \| METHOD | DETAIL: FIELD \| CONSTR \| METHOD |  |


---
